# Supplementary material for: A COVID-19 Silver Lining—Decline in Antibiotic Resistance in Ischemic Leg Ulcers during the Pandemic: A 6-Year Retrospective Study from a Regional Tertiary Hospital (2017–2022)
Source: Antibiotics (Basel). 2023 Dec 29;13(1):35. doi: 10.3390/antibiotics13010035 (PMC10812686; doi:10.3390/antibiotics13010035)
Supplement: Supplementary file 1 [file antibiotics-13-00035-s001.zip › antibiotics-2714416-supplementary.pdf]

# Supplementary Table S1

List of antibiotics and their respective classes against which gram-positive and gram-negative bacteria were tested for resistance in our study.

| Antibiotic class | Antibiotics | Gram-positive | Gram-negative |
|---|---|---|---|
| Aminoglycosides | Gentamicin | ✓ | ✓ |
| | Amikacin | × | ✓ |
| Cephalosporins | Cefotaxime | × | ✓ |
| | Ceftriaxone | × | ✓ |
| | Cefepime | × | ✓ |
| | Ceftazidime | × | ✓ |
| Fluoroquinolones | Ciprofloxacin | ✓ | ✓ |
| | Levofloxacin | ✓ | ✓ |
| | Moxifloxacin | ✓ | × |
| Macrolides | Erythromycin | ✓ | × |
| Penicillins | Penicillin G | ✓ | × |
| | Amoxicillin | × | ✓ |
| | Ampicillin | ✓ | ✓ |
| | Oxacillin | ✓ | × |
| | Piperacillin/tazobactam [A] | × | ✓ |
| Other | Clindamycin | ✓ | × |
| | Fusidic acid | ✓ | × |
| | Trimethoprim/sulfamethoxazole [A] | ✓ | ✓ |
| | Vancomycin | ✓ | × |
| | Carbapenems | × | ✓ |

[A] Antibiotics used in combination due to their synergistic effect.

# Supplementary Table S2

Antibiotic resistance and susceptibility of gram-positive bacteria isolated from leg ulcers in patients admitted to hospital with CLTI over the six-year study period (2017–2022).

| Pathogens n (%) | Overall | | Oxacillin | | Ampicillin | | Fusidic acid | | Clindamycin | | Erythromycin | | Trimethoprim / Sulfamethoxazole [A] | | Penicillin | | Aminoglycoside | | Fluoroquinolones | | Vancomycin | |
|---|---|---|---|---|---|---|---|---|---|---|---|---|---|---|---|---|---|---|---|---|---|---|
| | S | R | S | R | S | R | S | R | S | R | S | R | S | R | S | R | S | R | S | R | S | R |
| *Staphylococcus aureus* (*n*=19; 40%) | 16 (85%) | 3 (16%) | 18 (95%) | 1 (5%) | ND | | 18 (95%) | 1 (5%) | 17 (89%) | 2 (11%) | 17 (89%) | 2 (11%) | 18 (95%) | 1 (5%) | ND | | 19 (100%) | – | 19 (100%) | – | 19 (100%) | – |
| *Enterococcus* spp. (*E. faecalis, E. faecium, E. avium*) (*n*=17; 36%) | 11 (66%) | 6 (35%) | ND | | 15 (88%) | 2 (12%) | ND | | ND | | ND | | ND | | 15 (88%) | 2 (12%) | 13 (76%) | 4 (24%) | 17 (100%) | – | 16 (94%) | 1 (6%) |
| *β*- hemolytic Streptococci (*n*=7; 15%) | 7 (100%) | – | ND | | 7 (100%) | – | ND | | 7 (100%) | – | 7 (100%) | – | ND | | 7 (100%) | – | 7 (100%) | – | 7 (100%) | – | 7 (100%) | – |
| *Staphylococcus epidermidis* (*n*=2; 4%) | – | 2 (100%) | – | 2 (100%) | ND | | ND | | – | 2 (100%) | – | 2 (100%) | – | 2 (100%) | ND | | 1 (50%) | 1 (50%) | 1 (50%) | 1 (50%) | 2 (100%) | – |
| *α*-hemolytic Streptococci (*n*=1; 2%) | – | 1 (100%) | ND | | ND | | ND | | – | 1 (100%) | – | 1 (100%) | ND | | ND | | ND | | ND | | 1 (100%) | – |
| *Staphylococcus haemolyticus* (*n*=1; 2%) | 1 (100%) | – | 1 (100%) | – | ND | | ND | | 1 (100%) | – | 1 (100%) | – | 1 (100%) | – | ND | | 1 (100%) | – | 1 (100%) | – | 1 (100%) | – |
| Total (*n*=47) | 35 (75%) | 12 (26%) | 19 (86%) | 3 (14%) | 22 (92%) | 2 (8%) | 18 (95%) | 1 (5%) | 25 (83%) | 5 (17%) | 25 (83%) | 5 (17%) | 19 (84%) | 3 (16%) | 22 (92%) | 2 (8%) | 41 (89%) | 5 (11%) | 45 (98%) | 1 (2%) | 46 (98%) | 1 (2%) |

CLTI, chronic limb-threatening ischemia; ND, susceptibility testing was not performed; S, susceptible (in blue); R, resistant (in red).
Data within cells on the same row are *n* (%) for the number and proportion of isolated pathogens of a bacterial taxon tested against a particular antibiotic (columns).
[A] Antibiotics used in combination due to their synergistic effect.

# Supplementary Table S3

Antibiotic resistance and susceptibility of gram-negative bacteria isolated from leg ulcers in patients admitted to hospital with CLTI over the six-year study period (2017–2022).

| Bacterial taxa | Overall | | Carbapenems | | Cephalosporins | | Piperacillin / Tazobactam [A] | | Fluoroquinolones | | Aminoglycosides | | Ampicillin | | Amoxicillin / clavulanate [A] | | Trimethoprim / Sulfamethoxazole [A] | |
|---|---|---|---|---|---|---|---|---|---|---|---|---|---|---|---|---|---|---|
| | S | R | S | R | S | R | S | R | S | R | S | R | S | R | S | R | S | R |
| *Escherichia coli* (*n*=27; 15%) | 4 (15%) | 23 (85%) | 26 (96%) | 1 (4%) | 16 (59%) | 11 (41%) | 26 (96%) | 1 (4%) | 8 (30%) | 19 (70%) | 23 (85%) | 4 (15%) | 24 (88%) | 3 (11%) | 25 (93%) | 2 (7%) | 15 (56%) | 12 (44%) |
| *Pseudomonas aeruginosa* (*n*=26; 15%) | 24 (92%) | 2 (8%) | 24 (92%) | 2 (8%) | 25 (96%) | 1 (4%) | 26 (100%) | – | 25 (96%) | 1 (4%) | 25 (96%) | 1 (4%) | ND | | ND | | ND | |
| *Proteus mirabilis* (*n*=17); 10%) | 8 (47%) | 9 (53%) | 17 (100%) | – | 11 (65%) | 6 (35%) | 17 (100%) | – | 10 (53%) | 7 (47%) | 15 (87%) | 2 (12%) | 15 (87%) | 2 (12%) | 15 (87%) | 2 (12%) | 8 (47%) | 9 (53%) |
| *Citrobacter freundii* (*n*=16; 9%) | 8 (50%) | 8 (50%) | 15 (94%) | 1 (6%) | 14 (88%) | 2 (12%) | 13 (81%) | 3 (19%) | 14 (88%) | 2 (12%) | 15 (94%) | 1 (6%) | ND | | 10 (63%) | 6 (37%) | 12 (75%) | 4 (25%) |
| *Klebsiella pneumoniae* (*n*=15; 8%) | 5 (33%) | 10 (67%) | 14 (94%) | 1 (7%) | 9 (60%) | 6 (40%) | 13 (87%) | 2 (13%) | 6 (40%) | 9 (60%) | 15 (100%) | – | 14 (93%) | 1 (7%) | 11 (73%) | 4 (27%) | 6 (40%) | 9 (60%) |
| *Morganella morganii* (*n*=13; 7%) | 2 (15%) | 11 (85%) | 13 (100%) | – | 11 (85%) | 2 (15%) | 13 (100%) | – | 11 (85%) | 2 (15%) | 13 (100%) | – | 11 (85%) | 2 (15%) | 3 (23%) | 10 (77%) | 12 (93%) | 1 (7%) |
| *Stenotrophomonas maltophila* (*n*=9; 5%) | 6 (67%) | 3 (33%) | ND | | 8 (89%) | 1 (11%) | ND | | 7 (78%) | 2 (22%) | ND | | ND | | ND | | 9 (100%) | – |
| *Serratia marcescens* (*n*=8; 4%) | 2 (25%) | 6 (75%) | 8 (100%) | – | 8 (100%) | – | 8 (100%) | – | 8 (100%) | – | 8 (100%) | – | ND | | 2 (25%) | 6 (75%) | 8 (100%) | – |
| *Proteus vulgaris* (*n*=7; 4%) | 4 (57%) | 3 (43%) | 5 (71%) | 2 (29%) | 6 (86%) | 1 (14%) | 7 (100%) | – | 6 (86%) | 1 (14%) | 6 (86%) | 1 (14%) | 6 (86%) | 1 (14%) | 7 (100%) | – | 6 (86%) | 1 (14%) |
| *Enterobacter cloacae* (*n*=6; 3%) | 3 (50%) | 3 (50%) | 6 (100%) | – | 6 (100%) | – | 6 (100%) | – | 5 (83%) | 1 (17%) | 6 (100%) | – | ND | | 4 (67%) | 2 (33%) | 5 (83%) | 1 (17%) |
| *Klebsiella oxytoca* (*n*=6; 3%) | 4 (67%) | 2 (33%) | 6 (100%) | – | 5 (83%) | 1 (17%) | 6 (100%) | – | 4 (67%) | 2 (33%) | 6 (100%) | – | ND | | 6 (100%) | – | 5 (83%) | 1 (17%) |
| *Acinetobacter baumannii* (*n*=4; 2%) | 2 (50%) | 2 (50%) | 2 (50%) | 2 (50%) | 4 (100%) | – | 2 (50%) | 2 (50%) | 2 (50%) | 2 (50%) | 2 (50%) | 2 (50%) | 3 (75%) | 1 (25%) | ND | | 4 (100%) | – |
| Nonfermenting gram negative bacilli (*n*=4; 2%) | 3 (75%) | 1 (25%) | 4 (100%) | – | 4 (100%) | – | 4 (100%) | – | 3 (75%) | 1 (25%) | 3 (75%) | 1 (25%) | ND | | ND | | 4 (100%) | – |
| Others (*n*=20; 11%) [B] | 9 (40%) | 11 (60%) | 18 (90%) | 2 (10%) | 19 (95%) | 1 (5%) | 20 (100%) | – | 16 (80%) | 4 (20%) | 18 (90%) | 2 (10%) | 8 (89%) | 1 (11%) | 12 (63%) | 7 (37%) | 19 (100%) | – |
| Total (*n*=178) | 84 (47%) | 94 (53%) | 157 (93%) | 11 (7%) | 146 (82%) | 32 (18%) | 161 (95%) | 8 (5%) | 125 (70%) | 53 (30%) | 155 (92%) | 14 (8%) | 81 (88%) | 11 (12%) | 95 (71%) | 39 (29%) | 113 (75%) | 38 (25%) |

CLTI, chronic limb-threatening ischemia; ND, susceptibility testing was not performed; S, susceptible (in blue); R, resistant (in red). Data within cells on the same row are n (%) for the number and proportion of isolated pathogens of a bacterial taxon tested against a particular antibiotic (columns). [A] Antibiotics used in combination due to their synergistic effect. [B] This group included bacteria with fewer than 3 isolates, namely *Achromobacter xylosoxidans*, *Acinetobacter johnsonii*, *Aeromonas hydrophila*, *Citrobacter koseri*, *Delftia acidovorans*, *Haemophilus parainfluenzae*, *Proteus penneri*, *Providencia rettgeri*, *Providencia stuartii*, and *Pseudomonas putida*.
